# The Examination of Urine

**Published:** 1907-08-17

**Authors:** 


### Points in Pathology.

### II.—THE EXAMINATION OF URINE (*continued*).

Casts may be classified as (1) epithelial, (2) granular, (3) mixed, (4) hyaline, (5) waxy, (6) red blood, (7) leucocytes, and (8) hæmoglobin. 1. Epithelial: Those are found in acute cases of nephritis, and come from the tubules of the kidney; they vary in length, but are easily told by the epithelial cells which make them up. The nuclei of the cells may often be visible, while in other cases they may be indistinct, and the cytoplasm show fatty or granular changes. 2. Granular: Those are more common in sub-acute and chronic renal mischief, and in some instances are just a further change of an epithelial cast, in others they represent granular changes in what was previously an amorphous cast. The granules vary in size, and fatty globules may also be present scattered amongst them. 3. Mixed: As an acute nephritis passes into a sub-acute phase a certain amount of granular change begins to appear in the epithelial casts, this often going so far that the cast appears almost entirely granular with only a few epithelial cells adhering to it here and there. This gives us the mixed granular and epithelial cast. 4. Hyaline: Those are pale, colourless, and transparent, so much so as often to be difficult of detection. Where they arise from or what they mean is doubtful. Probably they represent epithelial casts that have undergone hyaline degeneration. 5. Waxy: Those resemble the former somewhat, but are more easily seen, and are broader. What they represent is again not very clear, and they do not indicate specially that amyloid disease of the kidneys is present. 6. Red blood cells: Casts consisting entirely of red blood corpuscles may be met with; they are recognised by the yellowish-green colour of the individual cells. 7. Leucocytes: Those sometimes form casts of themselves, but more frequently they are mixed with some of the other forms already described. 8. Hæmoglobin: In black water fever casts composed of hæmoglobin alone may be met with; they can be recognised by their yellow colour, and there is usually plenty of free hæmoglobin in masses found as well.

### Pus.

A few white cells in a urine does not mean very much, but when the number becomes excessive and they die and degenerate we get the condition of pus. They can be very easily told microscopically, as they are just degenerated polymorphonuclear leucocytes. When in excess it generally means some bacterium is present to account for them, and they have already been noted under the bacteriological examination of the urine. Cystitis due to the bacillus coli, the gonococcus, and the tubercle bacillus will cause abundant pus. In gonorrhœa, of course, one must exclude urethral pus before determining that cystitis is present.

### Blood.

Hæmorrhage into any part of the urinary tract will result in red cells being found in the urine. Many different causes may give rise to such a condition—nephritis, calculus in the kidney, cancer of the kidney, calculus in the bladder, tumours of the bladder, and bilharzial disease being amongst the more common. Microscopically, especially if the urine be examined fresh, the red blood cells may be seen very little altered, and are told readily by their shape, size and greenish-yellow colour.

### Epithelium.

The character of the epithelial cells found in the urine will vary, this depending on what part of the tract they have come from. As a matter of fact, in actual practice it is very difficult to tell renal from vesical epithelium with any degree of certainty, and this is unfortunate from the diagnostic point of view. Squamous cells from the urethra of both sexes, and the vagina of females are easily told by their large size and characteristic shape. A few epithelial cells in the urine is quite a common occurrence; there must normally be a certain degree of shedding, and those cast off are naturally passed in the water. In pathological conditions they are more numerous, and in acute cases of nephritis they will be found in abundance packed together as casts or lying in groups or singly.

### Ova and Parasites.

These, though uncommon in England, are frequent enough in parts of the tropics, and as cases from time to time crop up, it may not be out of place to mention the commoner ones here. In urinary cases of bilharzial disease the characteristic eggs will be found in the urine. The main symptom of this malady is hæmaturia, and such an occurrence in anyone coming from Africa should always make one exclude the bilharzia before passing on to other causes. The last drops of urine passed should be collected separately, and then examined (without centrifuging, as this breaks up the shells) under a low power of the microscope. The eggs will then be seen, as they are comparatively large objects, often entangled in little shreds of mucus, or free among the pus cells and blood. Having found some, a $\frac{1}{6}$-in. objective may next be employed to seek for the characteristic spine, which is placed terminally at the end of the shell, and if some water is then run under the cover-glass to mingle with the urine a ciliated embryo (miracidium) may be observed to come out of some of the eggs and move actively about. The next parasite that may sometimes be found in urine is the embryonic form of the Filaria bancrofti, the microfilaria sanguinis homini, or, as it is also called, the microfilaria nocturna, which is met with in chyluria.

### Foreign Bodies.

Hairs, cotton wool, and fibres of different sorts may readily fall into urine that is standing exposed; they are of no importance except in so far as they may be mistaken by the uninitiated for casts, worms, or other things. Vomit, sputum, tobacco, and other extraneous material may also find their way into the urine, not so much, it is true, in hospitals, as in private, but still one has seen some very awkward mistakes made by the overlooking of those odd contaminations.

